# Cardiovascular risk stratification using chest CT in idiopathic inflammatory myopathies

**DOI:** 10.1093/rheumatology/keag566

**Published:** 2026-10-12

**Authors:** Kastriot Kastrati, Svitlana Pochepnia, Rade Drača, Florian Prayer, Ece Danisman, Daniel Mrak, Thomas M Hofbauer, Daniel Aletaha, Latika Gupta, Hector Chinoy, Helmut Prosch, Helga Lechner-Radner

**Affiliations:** Division of Rheumatology, Department of Internal Medicine III, Medical University of Vienna, Vienna, Austria; Department of Rheumatology, Oslo University Hospital, Oslo, Norway; Division of General and Paediatric Radiology, Department of Biomedical Imaging and Image-guided Therapy, Medical University of Vienna, Vienna, Austria; Division of Rheumatology, Department of Internal Medicine III, Medical University of Vienna, Vienna, Austria; Division of General and Paediatric Radiology, Department of Biomedical Imaging and Image-guided Therapy, Medical University of Vienna, Vienna, Austria; Imaging Verbund, Vienna, Austria; Division of Rheumatology, Department of Internal Medicine III, Medical University of Vienna, Vienna, Austria; Division of Rheumatology, Department of Internal Medicine III, Medical University of Vienna, Vienna, Austria; Department of Internal Medicine II, Division of Cardiology, Medical University of Vienna, Vienna, Austria; Division of Rheumatology, Department of Internal Medicine III, Medical University of Vienna, Vienna, Austria; Department of Rheumatology, Royal Wolverhampton Hospitals NHS Trust, Wolverhampton, UK; School of Infection, Inflammation and Immunology, College of Medicine and Health, University of Birmingham, Birmingham, UK; Department of Rheumatology, Salford Royal Hospital, Northern Care Alliance NHS Foundation Trust, Manchester Academic Health Science Centre, Salford, UK; Division of Musculoskeletal and Dermatological Sciences, Faculty of Biology, Medicine and Health, The University of Manchester, Manchester, UK; Division of General and Paediatric Radiology, Department of Biomedical Imaging and Image-guided Therapy, Medical University of Vienna, Vienna, Austria; Division of Rheumatology, Department of Internal Medicine III, Medical University of Vienna, Vienna, Austria

**Keywords:** idiopathic inflammatory myopathies, coronary artery calcification, chest computed tomography, cardiovascular risk stratification, cardiovascular disease

## Abstract

**Objective:**

To evaluate the diagnostic and prognostic value of incidentally detected coronary artery calcification (CAC) on routine chest computed tomography (CT) for cardiovascular risk stratification in patients with idiopathic inflammatory myopathies (IIM).

**Methods:**

In this single-centre cross-sectional study, adult patients with IIM who underwent non-cardiac, non-gated high-resolution chest CT (HRCT) were included. CAC was visually categorized as absent, mild, moderate or severe. Diagnostic performance for established coronary artery disease (CAD) and established cardiovascular (CV) events was assessed. Kaplan–Meier analyses evaluated CV death-free survival, and multivariable logistic regression identified factors associated with CAC. A matched systemic sclerosis (SSc) cohort served as validation cohort.

**Results:**

Among 234 patients with IIM (mean age 57.1 [15.9] years; 70.1% female), CAC was present in 43.2%. Higher CAC thresholds showed greater specificity for established CAD and prior CV events, whereas lower thresholds were more sensitive. CAC was associated with reduced CV death-free survival (log-rank *P* = 0.0095), with a stepwise decline across CAC severity categories. Findings were similar in the matched SSc cohort. Increasing CAC burden was associated with older age, smoking, higher CV risk burden, interstitial lung disease and higher disease activity. In adjusted analyses, high disease activity was independently associated with moderate CAC (odds ratio [OR] 4.01; 95% CI: 1.29, 12.50), while a higher number of concomitant DMARDs was associated with severe CAC (OR 2.55; 95% CI: 1.27, 5.11).

**Conclusion:**

Incidentally detected CAC on routine chest CT provides diagnostic and prognostic information in IIM and may represent a pragmatic approach to improve cardiovascular risk stratification in this high-risk population.

## Graphical abstract

**Graphical Abstract:**
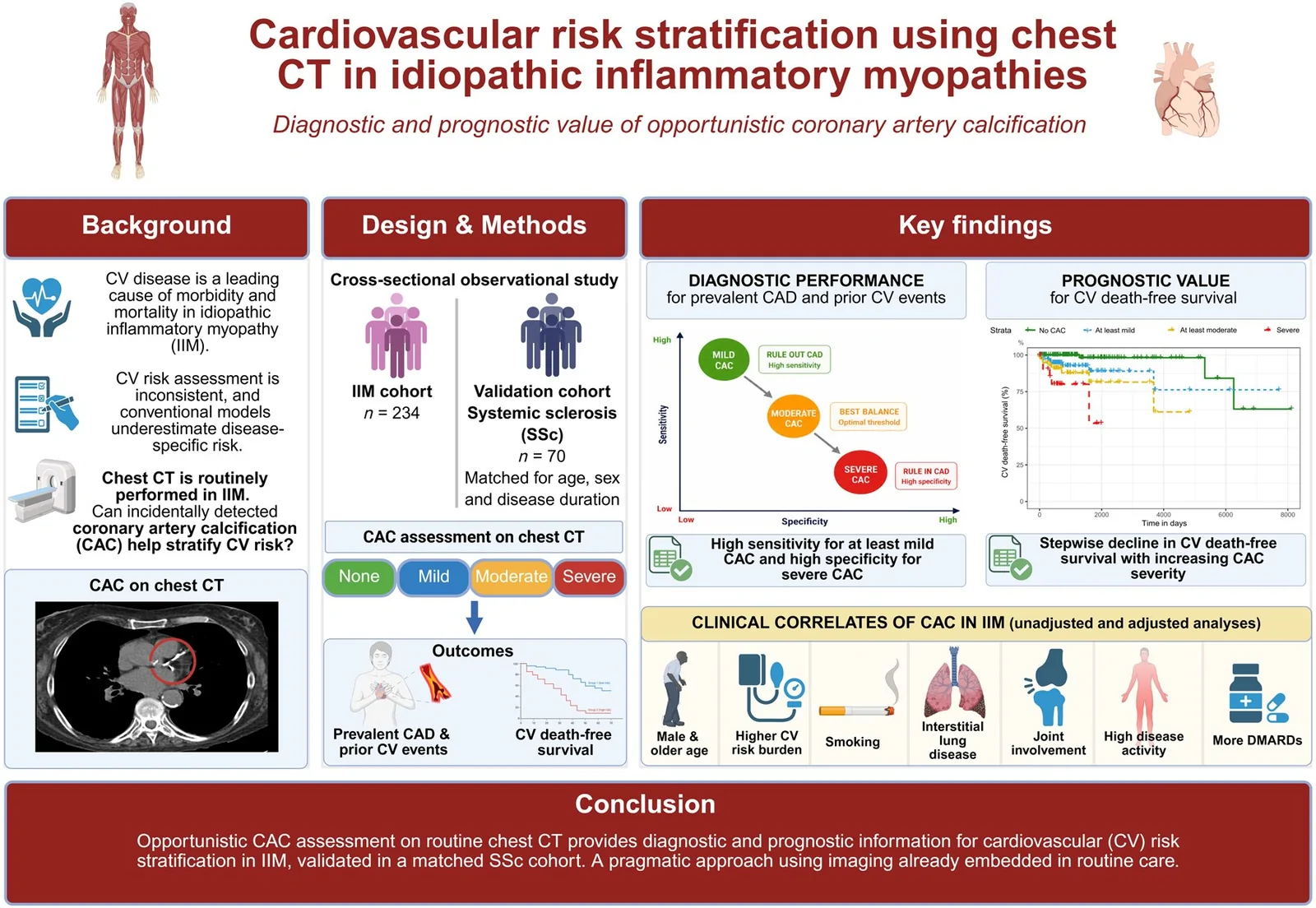

*Rheumatology* key messages

- Cardiovascular risk in idiopathic inflammatory myopathies (IIM) is high but remains insufficiently captured by conventional risk assessment tools.
- Routine chest CT enables incidental detection of coronary artery calcification (CAC) and provides clinically relevant cardiovascular risk stratification.
- Integrating CAC assessment into routine imaging may offer a simple and pragmatic strategy to improve cardiovascular risk stratification in high-risk populations such as IIM.

## Introduction

Idiopathic inflammatory myopathies (IIM) are a heterogeneous group of systemic autoimmune diseases characterized by chronic skeletal muscle inflammation and a wide spectrum of extramuscular manifestations, including interstitial lung disease (ILD), cutaneous and myocardial involvement. These systemic features substantially contribute to morbidity and mortality in affected individuals [1, 2].

Beyond these manifestations, cardiovascular disease (CVD) has increasingly been recognized as a major determinant of adverse outcomes in IIM [3–6]. Epidemiological studies indicate that patients with IIM carry an approximately 2- to 4-fold increased risk of coronary artery disease (CAD) and myocardial infarction compared with the general population [5]. This increased risk is thought to be driven by a combination of traditional cardiovascular risk factors and disease-related mechanisms such as chronic systemic inflammation, endothelial dysfunction and exposure to glucocorticoids or other immunosuppressive therapies [7–10].

CVD represents a substantial global economic burden, largely attributable to healthcare costs and loss of productivity associated with cardiovascular morbidity and mortality [9]. Despite increasing recognition of cardiovascular involvement in IIM, cardiovascular risk stratification in this population remains challenging. Established cardiovascular risk prediction tools, such as the Systematic Coronary Risk Evaluation (SCORE) and the Atherosclerotic Cardiovascular Disease (ASCVD) risk score, were developed for the general population and rely largely on traditional cardiovascular risk factors [11, 12]. As a result, these models may underestimate cardiovascular risk in chronic inflammatory diseases, including IIM [13], with clinically relevant risk potentially remaining under-recognized in routine care. Recent data indicate that a considerable proportion of patients with IIM fall into moderate-to-high cardiovascular risk categories, while cardiovascular risk assessment is not routinely performed in clinical practice [9]. Notably, commonly used risk prediction tools such as QRISK3 include rheumatoid arthritis and systemic lupus erythematosus, but not IIM, highlighting an unmet need for more accurate cardiovascular risk stratification for IIM in clinical practice [14].

In contemporary medicine, there is increasing recognition that the value derived from diagnostic procedures should be maximized beyond their primary indication. Resource-intensive investigations, such as computed tomography (CT), are often performed for specific clinical questions, yet may contain clinically relevant information beyond their primary purpose that remains underutilized in routine care. Leveraging such data may improve cost-effectiveness by increasing clinical value without additional resource use [15]. This concept is especially pertinent in systemic diseases such as IIM, where multimorbidity is common and the need to maximize the diagnostic yield of existing investigations is particularly high.

Against this background, chest CT represents a particularly relevant example in IIM, given its routine use in standard care. High-resolution CT (HRCT) of the chest is recommended by international societies, including the European Respiratory Society (ERS), the European Alliance of Associations for Rheumatology (EULAR) and the American College of Rheumatology/American College of Chest Physicians (ACR/CHEST), for the screening and assessment of ILD and for monitoring disease progression during follow-up in patients with established ILD [16–19]. In addition, chest CT is recommended in the context of malignancy screening, particularly in clinical constellations and IIM subtypes associated with increased risk of cancer [20].

Coronary artery calcification (CAC) detected on CT is a well-established marker of coronary atherosclerosis and a strong predictor of cardiovascular events in the general population [21–23]. Given the frequent use of chest CT in patients with IIM for ILD evaluation and malignancy screening, these imaging studies may offer an opportunity to extract additional clinically relevant information beyond pulmonary and oncological assessment. Opportunistic evaluation of CAC on routine chest CT may represent a pragmatic and readily available approach to improve cardiovascular risk stratification in this population. However, the potential clinical utility of CAC assessment derived from routine chest CT in patients with IIM has not been systematically evaluated.

Therefore, the aim of this study was to assess whether incidentally detected CAC on routine chest CT provides clinically relevant diagnostic and prognostic information for cardiovascular risk stratification in patients with IIM.

## Methods

### Study design and participants

This single-centre observational cross-sectional study included patients with IIM treated at the Division of Rheumatology, Medical University of Vienna, Austria. Patients were eligible if they fulfilled the Bohan and Peter criteria [24, 25] and, from 2017 onwards, the EULAR/ACR classification criteria for IIM [26]. Adult patients (≥18 years) were eligible if at least one non-cardiac, non-gated chest HRCT scan was available for review in the institutional picture archiving and communication system (PACS). The HRCT examinations were performed on institutional as well as external CT scanner platforms and used different non-gated acquisition protocols with varying slice thicknesses and reconstruction parameters. Patients without available imaging were excluded. This study was approved by the Ethics Committee of the Medical University of Vienna (identification no. 1833/2024).

### Data collection

Clinical data were obtained from our prospectively maintained registry of adult patients with IIM [27, 28]. Demographic and clinical variables included age, sex, body mass index, disease duration, smoking status and pack-years, IIM subtype, serological profile including myositis-specific and myositis-associated autoantibodies, and the presence of muscular and extramuscular manifestations. Cardiovascular (CV) variables comprised established cardiovascular risk factors such as arterial hypertension, hyperlipidaemia, diabetes mellitus, diagnosed coronary artery disease (CAD), prior myocardial infarction and family history of cardiovascular disease. Established CAD was defined as diagnosis confirmed by a cardiologist based on objective evidence of obstructive CAD on coronary angiography, coronary CT angiography or myocardial perfusion imaging, and/or a history of coronary revascularization by percutaneous coronary intervention (PCI) with stent implantation or coronary artery bypass grafting (CABG). Diagnosis of CAD as well as occurrence of a prior myocardial infarction were verified by review of the corresponding electronic medical records, including cardiology reports and discharge summaries. Medication data covered current and prior immunosuppressive treatment, including glucocorticoids, disease-modifying antirheumatic drugs (DMARDs) and intravenous immunoglobulin (IVIg), as well as concomitant medication such as antihypertensive agents, anti-diabetic and lipid-lowering therapy. For all analyses, clinical and laboratory data were assessed at the time of HRCT acquisition.

Disease activity was categorized as remission, low-to-moderate activity or high disease activity based on the Myositis Disease Activity Core Set Measures, including physician global assessment, patient global assessment, manual muscle testing (MMT-8), muscle enzyme levels, the Health Assessment Questionnaire, and the Myositis Disease Activity Assessment Tool, to assess extramuscular disease activity [29]. In the absence of validated cut-offs for disease activity states in IIM, categories were assigned using a composite clinical assessment integrating these measures: remission was defined as the absence of clinically relevant disease activity, as assessed by stable or normal muscle enzyme levels, low physician and patient global activity assessments, no evidence of active muscular or extramuscular manifestations, and a prednisolone-equivalent glucocorticoid dose of ≤5 mg/day. Patients classified as being in remission were not required to be in drug-free remission; maintenance of DMARD therapy in stable dosage was permitted. High disease activity was defined by clinical evidence of ongoing active disease, reflected by at least one physician global, patient global or extramuscular global activity score >5 on a 0–10 visual analogue scale, muscle enzyme levels >3-fold above the laboratory-specific upper limit of normal and/or documented decline in MMT-8, and/or the need for treatment escalation, including a glucocorticoid dose of ≥10 mg/day (prednisolone-equivalent) or initiation, addition or switching of immunosuppressive therapy due to increased disease activity. All remaining patients were classified as having low-to-moderate disease activity.

### Coronary artery calcification assessment

HRCT scans were independently assessed by two experienced thoracic radiologists, who were blinded to clinical data. CAC was visually graded using a previously described and validated ordinal scoring method [23, 30]. Briefly, the entire native coronary artery system, including the left main, left anterior descending, left circumflex, and right coronary artery, was evaluated and CAC was categorized as absent, mild, moderate or severe (Fig. 1). Inter-reader agreement for CAC grading was assessed using the intraclass correlation coefficient (ICC).

**Figure 1:**
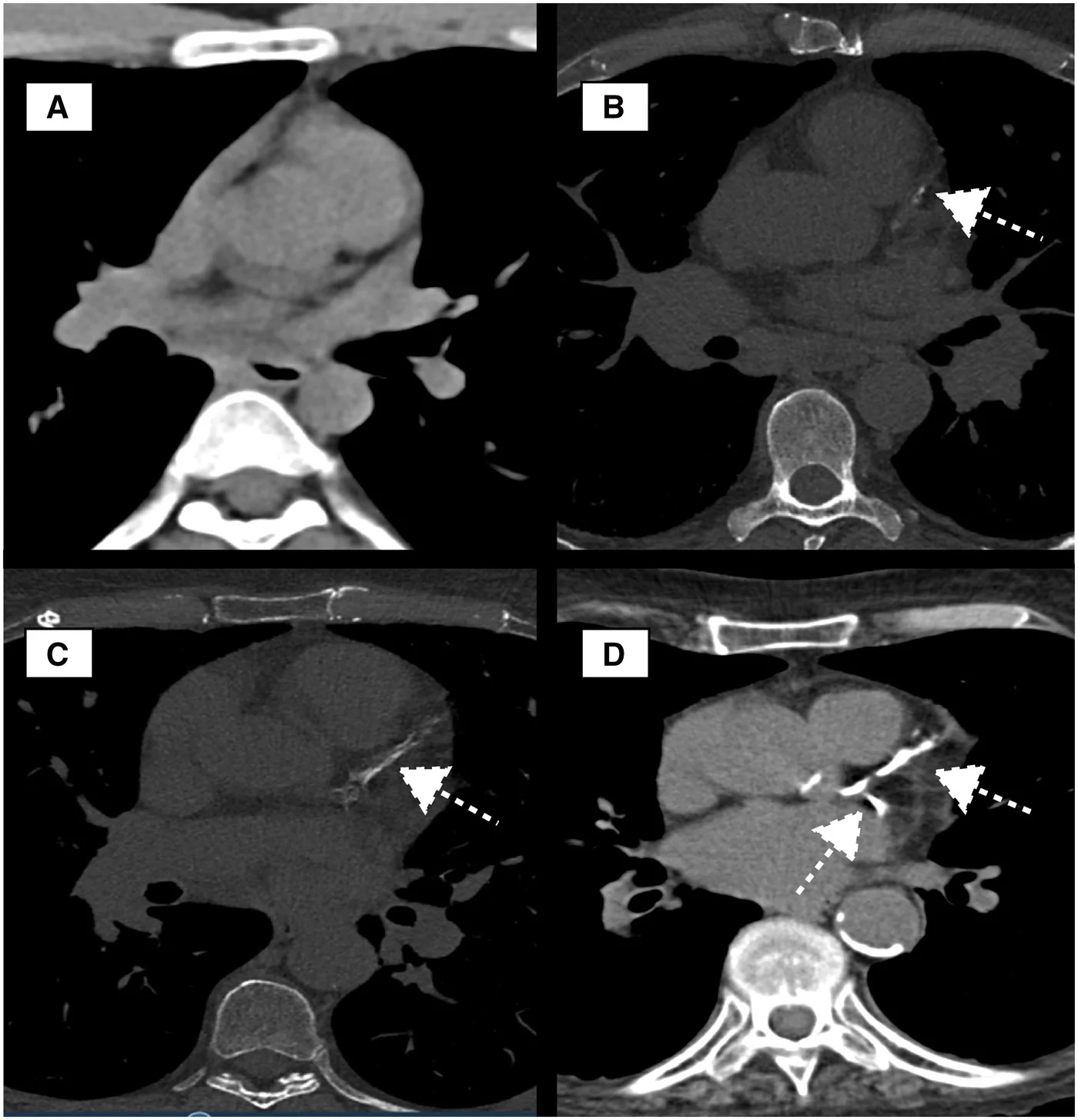
Visual assessment of coronary artery calcification (CAC) on routine chest CT in patients with idiopathic inflammatory myopathies (IIM). Representative axial CT images are shown for the four CAC categories used in this study: (**A**) absent CAC, (**B**) mild CAC, (**C**) moderate CAC, and (**D**) severe CAC. Arrows indicate calcified coronary segments

### Statistical analysis

Statistical analyses were performed using R version 4.5.1 (R Foundation for Statistical Computing, Vienna, Austria). The ggplot2 package was used for graphical displays. Categorical variables are presented as absolute frequencies and percentages, and continuous variables as mean and standard deviation or, where appropriate, median and interquartile range.

For the main analyses, patients were stratified according to CAC severity into no CAC, at least mild CAC, at least moderate CAC, and severe CAC. This hierarchical categorization was used to reflect increasing CAC burden and to evaluate the performance of different CAC severity thresholds, with lower thresholds favouring sensitivity and higher thresholds favouring specificity.

The diagnostic performance of CAC for identifying established CAD and prior cardiovascular CV events was assessed using sensitivity, specificity, positive predictive value (PPV) and negative predictive value (NPV), with the documented clinical diagnosis serving as the reference standard. CV events comprised non-fatal ST-segment elevation myocardial infarction (STEMI) and non-ST-segment elevation myocardial infarction (NSTEMI).

Time-to-event analyses were performed using the Kaplan–Meier method. Additional exploratory Cox proportional hazards regression analyses with separate adjustment for age, smoking status, disease activity and CV risk factor burden were performed in order to assess the association between CAC and CV death (results reported as hazard ratios [HRs] with 95% CIs). Event was defined as CV death, including sudden cardiac death and death due to acute myocardial infarction, heart failure, cardiovascular interventions or procedures, cardiovascular haemorrhage, or other cardiovascular causes, including peripheral artery disease; death due to pulmonary embolism was excluded. Follow-up time was calculated from the date of HRCT acquisition to the date of death or censoring at the last available assessment of vital status (31 December 2025). In addition, event rates per 100 person-years were calculated according to CAC category. To further examine the robustness and cross-disease applicability of the findings, sensitivity, specificity, PPV, NPV and Kaplan–Meier analyses for CV death were additionally evaluated in an age-, sex- and disease duration-matched cohort of patients with systemic sclerosis (SSc) treated at the same centre.

To assess the incremental prognostic value of visually assessed CAC beyond an established cardiovascular risk prediction model, QRISK3 [14] was calculated for all patients with IIM using clinical variables recorded at the timepoint of chest CT. Cox proportional hazards regression models were fitted for the composite end point of incident CV events (including non-fatal STEMI/NSTEMI) or CV death occurring after chest CT. Four models were evaluated: QRISK3 alone and QRISK3 combined with the predefined CAC thresholds (at least mild, at least moderate, and severe). We assessed the model discrimination by Harrell’s C-index, model fit by the Akaike information criterion (AIC) and the incremental prognostic value by likelihood-ratio tests in order to compare each CAC model with QRISK3 alone. In patients with IIM, associations between clinical variables and the presence or severity of CAC were examined using logistic regression models, with results reported as odds ratios (ORs) and 95% CIs. Multivariable models were adjusted for age, sex, disease duration and the presence of CV risk factor including smoking. Adjusted analyses were further repeated using Firth penalized logistic regression to reduce the potential impact of overfitting and small-sample bias [31]. Exploratory analyses were additionally performed to investigate associations between CAC severity and microvascular findings on nailfold capillaroscopy. These analyses were restricted to patients with capillaroscopic examinations performed within 6 months of HRCT. A two-sided *P*-value <0.05 was considered statistically significant.

## Results

### Patient characteristics

Of the 252 screened patients, 18 were excluded due to chest CT images not being available, leaving 234 patients with IIM for the final analysis. Patients were predominantly female (*n* = 164, 70.1%), with a mean age of 57.1 (15.9) years, mean body mass index of 25.1 (5.5) kg/m^2^ and mean disease duration of 5.6 (6.6) years. One hundred and forty-two (60.7%) never smoked and 42 (17.9%) patients were active smokers. The most frequent IIM subtype was dermatomyositis (*n* = 78, 33.3%), followed by overlap myositis (*n* = 61, 26.1%), antisynthetase syndrome (*n* = 50, 21.4%), immune-mediated necrotizing myopathy (*n* = 26, 11.1%), polymyositis (*n* = 15, 6.4%) and inclusion body myositis (*n* = 4, 1.7%). Muscle involvement was present in 162 patients (69.2%). The most common extramuscular manifestations were arthralgia (*n* = 141, 60.3%), ILD (*n* = 91, 38.9%), skin involvement (*n* = 91, 38.9%) and Raynaud’s phenomenon (*n* = 80, 34.2%) (Table 1).

**Table 1:** Clinical characteristics of patients with IIM at the time of chest CT acquisition.

Variable	Total (*n* = 234)
Demographics	
Female, *n* (%)	164 (70.1)
Age, mean (s.d.), years	57.1 (15.9)
BMI, mean (s.d.), kg/m^2^	25.1 (5.5)
Disease duration, mean (s.d.), years	5.6 (6.6)
Tobacco use	
Never, *n* (%)	142 (60.7)
Former, *n* (%)	50 (21.4)
Current, *n* (%)	42 (17.9)
Pack-years, mean (s.d.) (*n* = 92)	25.5 (19.1)
IIM subtype, *n* (%)	
Dermatomyositis	78 (33.3)
Polymyositis	15 (6.4)
Antisynthetase syndrome	50 (21.4)
Overlap myositis	61 (26.1)
Immune-mediated necrotizing myopathy	26 (11.1)
Inclusion body myositis	4 (1.7)
Clinical features, *n* (%)	
Muscle involvement	162 (69.2)
Interstitial lung disease	91 (38.9)
Skin involvement	91 (38.9)
Oesophageal involvement	50 (21.4)
Myocardial involvement	17 (7.3)
Arthralgia	141 (60.3)
Arthritis	64 (27.4)
Raynaud’s phenomenon	80 (34.2)
Malignancy	30 (12.8)
Disease activity, *n* (%)	
Remission	58 (24.8)
Low-to-moderate	140 (59.8)
High	36 (15.4)
CV risk factors, *n* (%)	
Number of CV risk factors	
0	44 (18.8)
1	65 (27.8)
2	58 (24.8)
≥3	67 (28.6)
Arterial hypertension	104 (44.4)
Hyperlipidaemia	127 (54.3)
Diabetes mellitus	23 (9.8)
Established coronary artery disease	57 (24.4)
History of myocardial infarction	26 (11.1)
Family history of cardiovascular disease	93 (41.2)
Autoantibodies, *n* (%)	
Antinuclear antibodies	113 (48.3)
Myositis-specific autoantibodies	102 (43.6)
Myositis-associated autoantibodies	98 (41.9)
Treatment	
Glucocorticoid use, *n* (%)	95 (40.6)
Prednisolone-equivalent dose, mean (s.d.), mg/day	10.3 (13.0)
Intravenous immunoglobulin, *n* (%)	42 (17.9)
Number of DMARDs, *n* (%)	
0	72 (30.8)
1	123 (52.6)
2	34 (14.5)
≥3	5 (2.1)

Abbreviations: BMI: body mass index; CT: computed tomography; CV: cardiovascular; DMARD: disease-modifying antirheumatic drug; IIM: idiopathic inflammatory myopathy.

At the time of chest CT being carried out, 58 patients (24.8%) were in remission, 140 (59.8%) had low-to-moderate disease activity and 36 (15.4%) had high disease activity. Cardiovascular disease and risk factors were common: 104 patients (44.4%) had arterial hypertension, 127 (54.3%) hyperlipidaemia, 23 (9.8%) diabetes mellitus, 57 (24.4%) established coronary artery disease, 26 (11.1%) prior myocardial infarction and 93 (41.2%) a family history of cardiovascular disease; overall, 67 patients (28.6%) had ≥3 cardiovascular risk factors. At the time of chest CT, 95 patients (40.6%) were receiving glucocorticoids (mean prednisolone-equivalent dose 10.3 (13.0) mg/day). Most patients received one DMARD (*n* = 123, 52.6%), whereas 72 (30.8%) received none, 34 (14.5%) two and 5 (2.1%) three or more; 42 (17.9%) received IVIg (Table 1). Detailed distributions of individual DMARDs and specific autoantibodies are shown in Supplementary Fig. S1.

### Diagnostic performance of chest CT-derived CAC

In the IIM cohort, 133 patients (56.8%) had no CAC, 41 (17.5%) had mild CAC, 38 (16.2%) had moderate CAC and 22 (9.4%) had severe CAC. Established coronary artery disease (CAD) was present in 57 patients, prior myocardial infarction in 26 and the combined end point of established CAD or prior CV event in 65 patients. Baseline clinical characteristics stratified by CAC grade are provided in Supplementary Table S1. Inter-reader agreement for CAC grading was high, with an ICC of 0.90 (95% CI: 0.88, 0.93).

The diagnostic performance of the different CAC thresholds for identifying established CAD, prior CV event and the combined outcome in patients with IIM is shown in Fig. 2A. For established CAD, lower CAC thresholds were associated with higher sensitivity and NPV, whereas higher CAC thresholds showed increasing specificity and PPV. Specifically, at least mild CAC yielded a sensitivity of 93.0%, specificity of 72.9%, PPV of 52.5% and NPV of 97.0%; for at least moderate CAC, the corresponding values were 71.9%, 89.3%, 68.3% and 90.8%; and for severe CAC, 31.6%, 97.7%, 81.8% and 81.6%, respectively. A similar pattern was observed for the combined outcome of established CAD or prior CV event, with sensitivity decreasing and specificity increasing across higher CAC categories: 90.8%, 75.1%, 58.4% and 95.5% for at least mild CAC; 70.8%, 91.7%, 76.7% and 89.1% for at least moderate CAC; and 29.2%, 98.2%, 86.4% and 78.3% for severe CAC.

**Figure 2:**
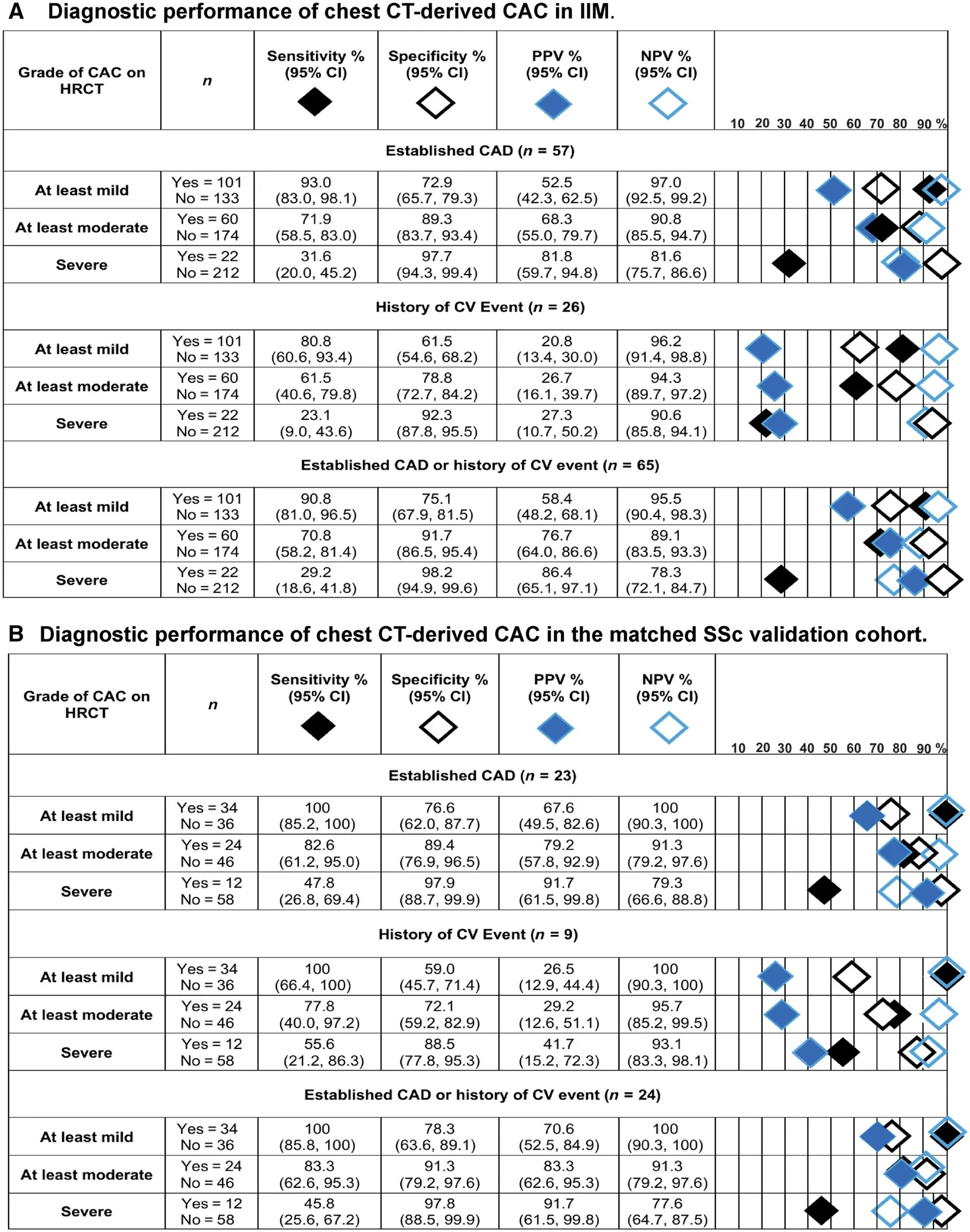
Diagnostic performance of chest CT-derived coronary artery calcification (CAC) for identifying established coronary artery disease and prior cardiovascular events in patients with idiopathic inflammatory myopathies (IIM) and in the matched systemic sclerosis (SSc) validation cohort. (**A**) Sensitivity, specificity, positive predictive value (PPV) and negative predictive value (NPV) of different CAC thresholds for identifying established coronary artery disease (CAD), prior cardiovascular (CV) events, and the combined end point of established CAD or prior CV event in patients with IIM. (**B**) Sensitivity, specificity, PPV and NPV of different CAC thresholds for identifying established CAD, prior CV events, and the combined end point in the matched SSc validation cohort

For validation, the same analyses were performed in 70 patients with SSc, matched for age, sex and disease duration (mean age 53.8 [14.0] years, 49 female patients [70.0%], mean disease duration 6.29 [5.62] years). In this cohort, established CAD was present in 23 patients, prior CV event in 9 and the combined outcome in 24 patients (Fig. 2B). For the combined outcome of established CAD or prior CV event, the same gradient was observed, with at least mild CAC showing the highest sensitivity (100%) and NPV (100%), whereas severe CAC showed the highest specificity (97.9%) and PPV (91.7%). For at least moderate CAC, sensitivity, specificity, PPV and NPV were 82.6%, 89.4%, 79.2% and 91.3%, respectively.

### CAC and CV-related mortality

In the IIM cohort, Kaplan–Meier analysis of CV death-free survival based on the absence or presence of CAC is shown in Fig. 3A and demonstrated a significant difference between groups (log-rank *P* = 0.0095). Exploratory Cox proportional hazards analysis demonstrated an unadjusted association between CAC and CV death (HR 5.05; 95% CI: 1.31, 19.45; *P *= 0.019). The association remained largely unchanged after separate adjustment for smoking (HR 5.16; 95% CI: 1.35, 19.76; *P* = 0.017), disease activity (HR 5.25; 95% CI: 1.34, 20.61; *P* = 0.018) and CV risk factor burden (HR 5.42; 95% CI: 1.27, 23.12; *P* = 0.022), whereas the association was not statistically significant after adjustment for age (HR 2.64; 95% CI: 0.58, 11.99; *P* = 0.208).

**Figure 3:**
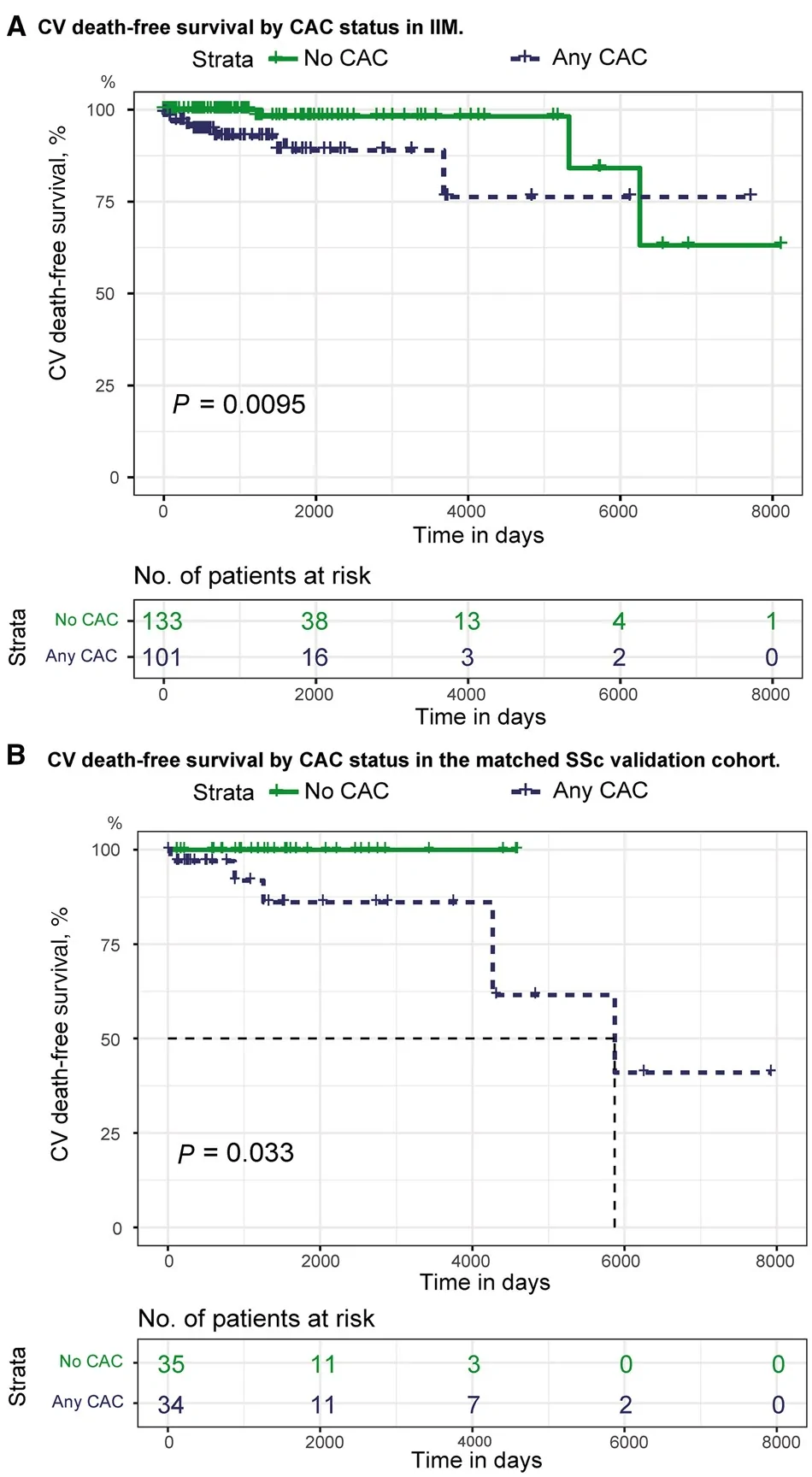
Cardiovascular (CV) death-free survival according to the absence or presence of coronary artery calcification (CAC) on chest CT. (**A**) Kaplan–Meier analysis of CV death-free survival in patients with idiopathic inflammatory myopathies (IIM) stratified by absence *vs* presence of CAC. (**B**) Kaplan–Meier analysis of CV death-free survival in the matched systemic sclerosis (SSc) validation cohort stratified by absence *vs* presence of CAC

In the matched SSc validation cohort, Kaplan–Meier analysis according to CAC status likewise showed a significant difference in CV death-free survival (log-rank *P* = 0.033; Fig. 3B). Additional stratification of the IIM cohort according to CAC severity showed a stepwise decrease in CV death-free survival with increasing CAC severity, with the poorest survival observed in patients with severe CAC (Fig. 4). A corresponding stratified analysis was not performed in the SSc cohort because of the low frequency of CV death events.

**Figure 4:**
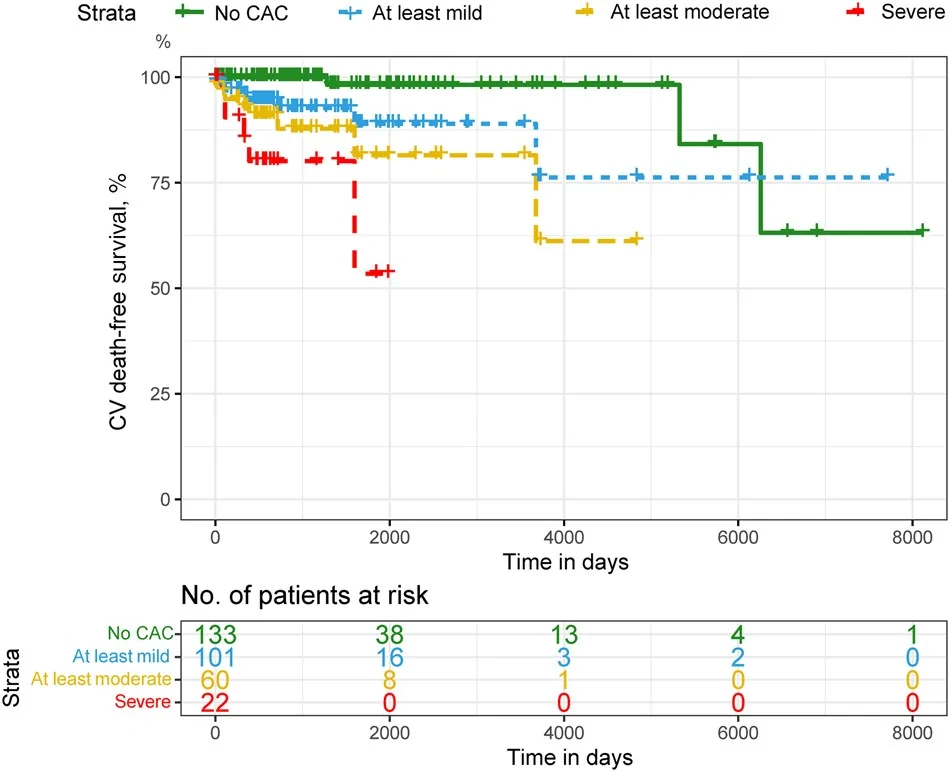
Kaplan–Meier analysis of cardiovascular death-free survival in patients with idiopathic inflammatory myopathies (IIM) stratified by coronary artery calcification (CAC) severity on chest CT. Patients were grouped as no CAC (green), at least mild CAC (blue), at least moderate CAC (yellow), and severe CAC (red)

### Prognostic value of CAC beyond QRISK3

Compared with QRISK3 alone, the addition of visually assessed CAC improved model discrimination and model fit across all predefined CAC thresholds. Detailed results of all Cox regression models are shown in Supplementary Table S2.

### Clinical correlates of CAC in IIM

In unadjusted analyses, increasing CAC burden was primarily linked to age, smoking, cardiovascular risk burden and selected clinical manifestations. Older age was associated with all CAC thresholds, with ORs of 1.10 (95% CI: 1.07, 1.13) for at least mild CAC, 1.10 (1.06, 1.13) for at least moderate CAC and 1.08 (1.04, 1.12) for severe CAC. Former smoking was likewise associated with increasing CAC severity, particularly severe CAC (OR 4.19; 95% CI: 1.55, 11.32). Among disease-related variables, ILD was associated with at least mild CAC (OR 2.20; 1.29, 3.76), at least moderate CAC (OR 2.23; 1.23, 4.04) and severe CAC (OR 2.48; 1.01, 6.07), whereas arthralgia and arthritis showed particularly strong associations, most notably for mild and moderate CAC (arthritis: OR 7.41 [3.82, 14.40] and 5.07 [2.69, 9.58], respectively). A higher burden of cardiovascular risk factors was consistently associated with CAC across all thresholds (at least mild OR 2.39 [1.84, 3.11]; severe OR 2.32 [1.59, 3.39]) (Supplementary Fig. S2; Supplementary Table S3). In the subset with nailfold capillaroscopy available within 6 months of the respective HRCT, no consistent associations were observed between CAC severity and microvascular abnormalities on capillaroscopy, except for an association between capillary tortuosities and severe CAC (OR 5.26; 95% CI: 1.06, 26.22) (Supplementary Fig. S3).

After adjustment for age, sex, presence of a traditional CV risk factor including smoking, and disease duration, the strongest associations persisted for joint involvement and high disease activity (Fig. 5). Severe CAC was independently associated with a higher number of concomitant DMARDs (OR 2.55 [95% CI: 1.27, 5.11] per additional DMARD) (Fig. 5; Supplementary Table S4). Analyses using Firth penalized logistic regression showed consistent results (Supplementary Table S5). When expressed as events per 100 person-years, CV-related death rates increased across CAC categories, from 0.50 (95% CI: 0.10, 1.48) in patients without CAC to 2.41 (1.04, 4.75) for at least mild CAC, 4.34 (1.87, 8.55) for at least moderate CAC and 11.85 (3.85, 27.65) for severe CAC (Supplementary Fig. S4; Supplementary Table S6).

**Figure 5:**
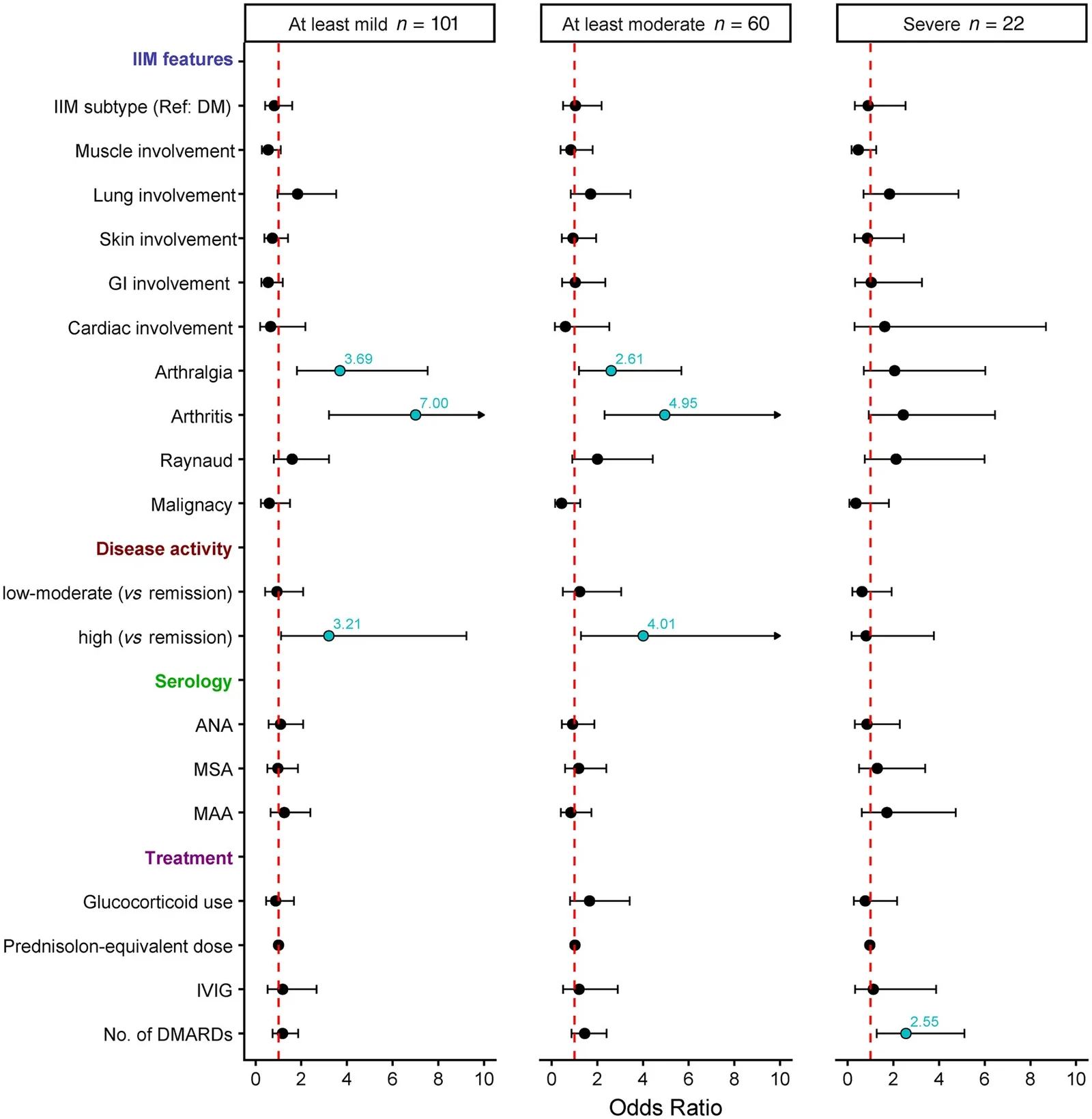
Clinical factors associated with the presence and severity of coronary artery calcification (CAC) assessed on chest CT in patients with idiopathic inflammatory myopathies (IIM). Forest plots show ORs with 95% CIs for clinical, serological, disease-related, and treatment-related variables associated with at least mild, at least moderate, and severe CAC on chest CT, each compared with no CAC as the reference category. Analyses were performed using multivariable logistic regression. Vertical dashed lines indicate OR = 1. Variables with statistically significant associations (*P* < 0.05) are highlighted in turquoise. Abbreviations: CAC: coronary artery calcification; DMARDs: disease-modifying antirheumatic drugs; GI: gastrointestinal; IVIg: intravenous immunoglobulin; MAA: myositis-associated antibodies; MSA: myositis-specific antibodies; OR: odds ratio

## Discussion

In this single-centre cohort of patients with IIM, CAC assessed on routine chest CT showed clinically relevant diagnostic and prognostic utility. Our findings support that CAC derived from routine chest CT provides meaningful cardiovascular risk information in a real-world setting. Different CAC thresholds identified distinct risk profiles. Notably, the at least mild CAC threshold showed high sensitivity and NPV, supporting its potential role as a rule-out or screening-oriented threshold, whereas higher CAC categories provided greater specificity. In addition, CAC was associated with reduced CV death-free survival, with a clear stepwise gradient across CAC severity categories. This graded relationship may be clinically useful, as increasing CAC burden was associated with a progressively higher risk of adverse cardiovascular outcomes. The overall performance of this method was reliable and could be reproduced in an age-, sex- and disease duration-matched SSc validation cohort, supporting the robustness of the approach.

Our findings may be clinically relevant as CV disease is increasingly recognized as a major source of morbidity and mortality in IIM, alongside lung disease and malignancy [1, 3, 9, 20]. Population-based studies and meta-analyses have shown that patients with IIM carry a significantly increased burden of coronary disease and myocardial infarction compared with the general population, with risk estimates commonly in the 2- to 4-fold range [3]. At the same time, CV risk assessment in IIM remains suboptimal. Conventional tools such as SCORE-, ASCVD-, or other population-derived algorithms underestimate CV risk in inflammatory rheumatic diseases, as they do not take into account disease-specific drivers such as cumulative burden of inflammation, glucocorticoid exposure or disease activity [9]. Our findings address this gap by suggesting that estimation of cardiovascular risk can be derived from imaging already obtained for other clinical indications. Rather than relying solely on risk calculators not validated for IIM, incidentally detected CAC on routine chest CT may provide a pragmatic and readily available approach to risk stratification. A major strength of our approach may therefore be its opportunistic nature. In contemporary myositis care, chest CT is frequently obtained for ILD assessment, ILD follow-up, and, particularly in dermatomyositis, malignancy screening. Against this background, opportunistic CAC assessment on routine chest CT may offer a pragmatic way to derive additional cardiovascular information from imaging already performed for other clinical indications. In contrast to dedicated ECG-gated non-contrast cardiac CT, which remains the reference standard for Agatston-based quantification, this approach does not provide formal calcium scoring but may still help identify patients with a higher atherosclerotic burden.

Our additional analyses suggest that visually assessed CAC may provide prognostic information beyond the established QRISK3 cardiovascular risk score. Compared with QRISK3 alone, the addition of CAC improved model discrimination and model fit across the predefined CAC thresholds. This may support the idea that CAC assessment may complement conventional CV risk stratification in patients with IIM. Notably, CAC alone was also associated with the composite outcome (HR 10.15; 95% CI: 2.84, 36.28). However, these findings should be interpreted with caution, as only 17 outcome events occurred during follow-up. Also, the hazard ratio estimates showed wide confidence intervals, which is associated with the small number of outcome events. In addition, QRISK3 was originally developed to estimate the 10-year risk of a first cardiovascular event, whereas the present analysis evaluated a composite end point (CV events or CV death) over a mean follow-up of 4.64 (4.60) years. Therefore, these analyses should be regarded as exploratory.

The present findings suggest that the patients at highest risk were those with older age, greater conventional CV risk burden, smoking history and more advanced CAC severity. In addition, ILD, joint involvement and high disease activity showed consistent associations with CAC in unadjusted and, in part, adjusted analyses. Severe CAC was also associated with a higher number of concomitant DMARDs, although this finding should be interpreted cautiously, as it likely reflects treatment escalation in patients with more severe or refractory disease rather than a direct adverse cardiovascular effect of DMARD exposure itself. This pattern is noteworthy because it places CAC at the intersection of traditional and disease-related CV risk. In practical terms, patients combining inflammatory disease burden with conventional risk enrichment may represent the subgroup most likely to benefit from CAC-informed risk stratification. The validation in the matched SSc cohort is an important feature of this study, as it provides cross-disease support for the observed associations. The reproducibility of findings across IIM and SSc suggests that opportunistic CAC assessment on routine chest CT is not only a cohort-specific observation but may reflect a broader principle applicable to systemic autoimmune disease.

Opportunistic CAC assessment is not a substitute for dedicated cardiac CT when precise quantification is required, and it does not capture non-calcified plaque, plaque morphology or luminal stenosis the way coronary CT angiography can. Dedicated gated cardiac CT remains the more standardized and technically refined method for calcium scoring, and coronary CT angiography provides additional anatomical information that routine chest CT cannot offer. However, CAC assessment on chest CT may help identify patients who may benefit from further cardiovascular work-up, using imaging already obtained in standard IIM care.

Strengths of this study include the evaluation of a clinically available, low-threshold imaging marker in a relatively large real-world IIM cohort, blinded CAC assessment with high inter-reader agreement, the integration of diagnostic and survival analyses, and replication of the main findings in a matched SSc cohort. At the same time, limitations should be recognized. The study was single-centre, and although the matched SSc cohort supports the reproducibility of the observed associations, it should be considered a cross-disease replication rather than a formal external validation. The relatively small sample size and limited number of CV deaths in this cohort precluded further comparative analyses, which may reduce the reliability of the findings. Although only a small number of patients with IIM were excluded because no HRCT examination was available, we nevertheless need to account for the possibility of selection bias. In addition, the limited number of CV deaths, particularly in the SSc cohort, may reduce the stability of Kaplan–Meier estimates at later follow-up time points and limit the precision of long-term survival analyses. Also, visual CAC grading, while pragmatic and clinically attractive, is less granular than dedicated quantitative scoring. Furthermore, prior cardiovascular events and diagnosis of CAD were identified from clinical records rather than using a reference standard. As cardiac investigations were performed according to clinical indications rather than a standardized protocol, verification bias and differential ascertainment cannot be excluded. Finally, because the study design is observational, the data cannot determine whether CAC-guided management would improve outcomes.

Overall, our findings support opportunistic CAC assessment on routine chest CT as a feasible and clinically meaningful approach to maximize the clinical value of existing imaging in IIM. In a disease setting where CV risk is high, formal risk assessment remains inconsistent, and thoracic CT is already deeply embedded in routine care, this approach may help identify patients who warrant closer CV evaluation and more intensive preventive management. Future prospective studies should determine how CAC can be incorporated into cardiovascular work-up, preventive strategies and risk prediction models in IIM.

## Supplementary Material

keag566_Supplementary_Data

## Data Availability

All data relevant to this study are included in the article or provided in the online supplementary material. An anonymized minimal dataset can be made available to qualified researchers upon reasonable request and following approval of a study proposal by the corresponding author (helga.lechner-radner@meduniwien.ac.at) and the respective local ethics committees.
