# Supplementary material for: Cardiovascular risk stratification using chest CT in idiopathic inflammatory myopathies
Source: Rheumatology (Oxford). 2026 Oct 12;65(10):keag566. doi: 10.1093/rheumatology/keag566 (PMC13707383; doi:10.1093/rheumatology/keag566)
Supplement: Supplementary file 1 [file keag566_supplementary_data.pdf]

Cardiovascular risk stratification using chest CT in idiopathic inflammatory myopathies

Online Supplementary appendix

Table of contents

Supplementary Figure S1. Frequency distribution of myositis-specific and myositis-associated autoantibodies and immunomodulatory treatments in patients with idiopathic inflammatory myopathies……………………………………..3

Supplementary Table S1. Clinical characteristics of patients with IIM stratified by CAC grade at the time of chest CT acquisition ……..4

Supplementary Table S2. Additional prognostic value of visually assessed coronary artery calcification (CAC) beyond QRISK3…….5

Supplementary Figure S2. Unadjusted odds ratios for clinical correlates of coronary artery calcification (CAC) severity in patients with IIM ……..6

Supplementary Table S3. Unadjusted odds ratios for clinical correlates of CAC severity in patients with IIM ……….7

Supplementary Table S4. Adjusted odds ratios for clinical correlates of CAC severity in patients with IIM ………8

Supplementary Table S5. Adjusted odds ratios from Firth penalized logistic regression models for clinical correlates of CAC severity in patients with IIM……………………………………………………………………………………………9

Supplementary Figure S3. Associations between nailfold capillaroscopic abnormalities and coronary artery calcification (CAC) severity in patients with IIM……………………………………………………………………………………10

Supplementary Figure S4. CV-related death rates according to coronary artery calcification (CAC) category in patients with IIM.……..11

Supplementary Table S6. CV-related death according to coronary artery calcification (CAC) category, expressed as events per 100 person-years.………………………………………………………………………………………………………11

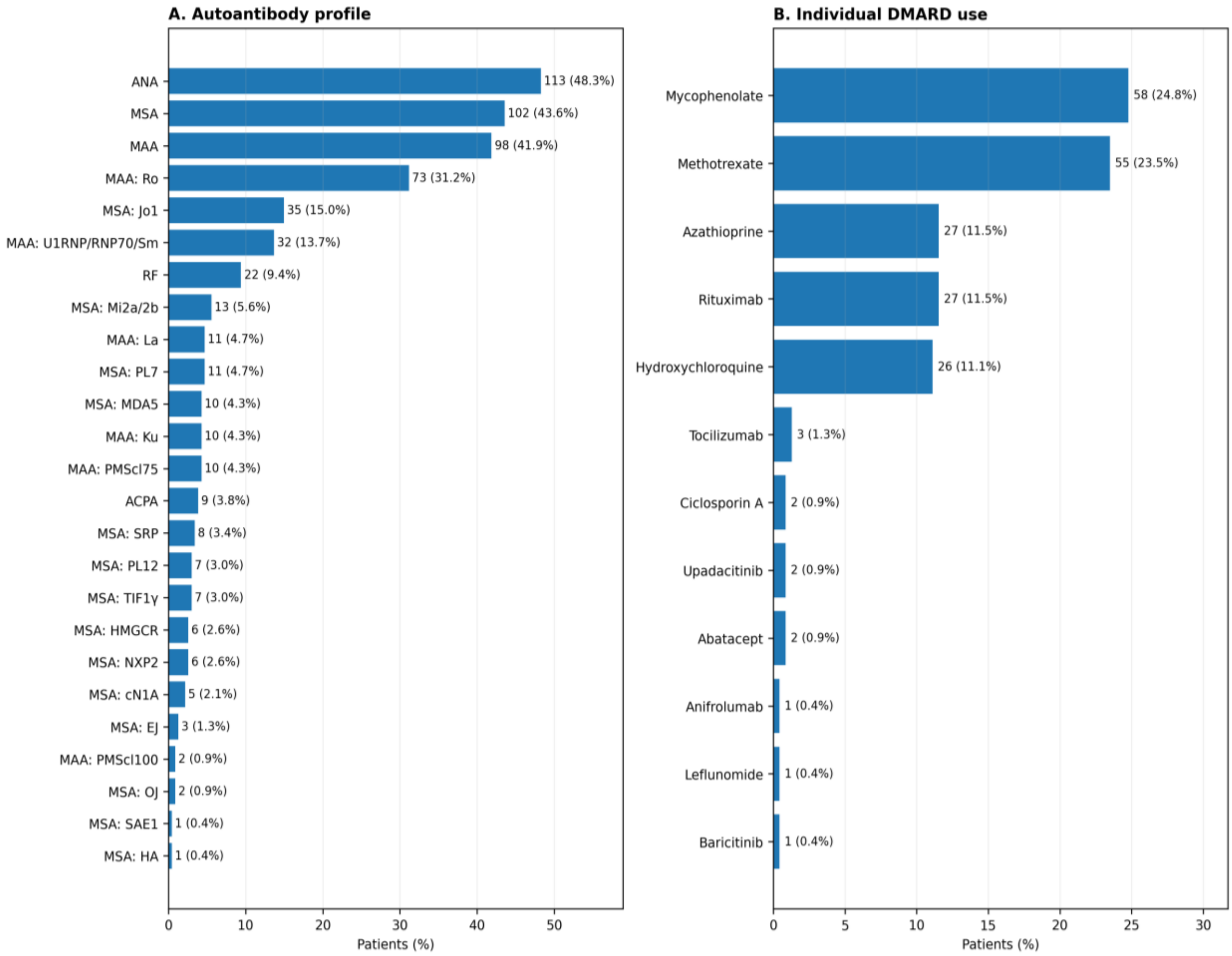

Supplementary Figure S1. Frequency distribution of myositis-specific (MSA) and myositis-associated autoantibodies (MAA) and immunomodulatory treatments in patients with IIM. Values are presented as numbers and percentages (%) of the overall cohort.

Variable	Absent CAC (n=133)	CAC mild (n=41)	CAC moderate (n=38)	CAC severe (n=22)	p value
**Sex**					
Female	101 (75.9%)	31 (75.6%)	22 (57.9%)	10 (45.5%)	<0.01
Male	32 (24.1%)	10 (24.4%)	16 (42.1%)	12 (54.5%)	
Age, years	49.6 ± 14.8	63.2 ± 12.4	68.9 ± 10.2	70.7 ± 9.7	<0.01
BMI, kg/m²	24.8 ± 4.7	24.8 ± 4.5	26.2 ± 8.5	25.7 ± 5.0	0.20
Disease duration, years	5.1 ± 6.4	6.5 ± 6.0	4.8 ± 7.7	6.6 ± 6.0	0.48
**Tobacco use**					<0.01
Never	89 (66.9%)	23 (56.1%)	22 (57.9%)	8 (36.4%)	
Former	21 (15.8%)	10 (24.4%)	9 (23.7%)	10 (45.5%)	
Current	23 (17.3%)	8 (19.5%)	7 (18.4%)	4 (18.2%)	
Pack-years (n=92)	21.3 ± 18.0	21.2 ± 14.8	33.1 ± 22.4	35.5 ± 19.1	<0.01
**IIM subtype**					
Dermatomyositis	46 (34.6%)	12 (29.3%)	13 (34.2%)	7 (31.8%)	0.79
Polymyositis	8 (6.0%)	2 (4.9%)	3 (7.9%)	2 (9.1%)	0.56
Antisynthetase syndrome	30 (22.6%)	7 (17.1%)	7 (18.4%)	6 (27.3%)	0.99
Overlap myositis	37 (27.8%)	14 (34.1%)	6 (15.8%)	4 (18.2%)	0.16
Immune-mediated necrotizing myopathy	11 (8.3%)	5 (12.2%)	7 (18.4%)	3 (13.6%)	0.12
Inclusion body myositis	1 (0.8%)	1 (2.4%)	2 (5.3%)	0 (0.0%)	1.00
**Clinical features**					
Muscle involvement	97 (72.9%)	25 (61.0%)	28 (73.7%)	12 (54.5%)	0.18
Interstitial lung disese	41 (30.8%)	18 (43.9%)	19 (50.0%)	13 (59.1%)	<0.01
Skin involvement	57 (42.9%)	14 (34.1%)	13 (34.2%)	7 (31.8%)	0.19
Esophageal involvement	30 (22.6%)	6 (14.6%)	9 (23.7%)	5 (22.7%)	0.99
Heart involvement	11 (8.3%)	3 (7.3%)	1 (2.6%)	2 (9.1%)	0.57
Arthralgia	68 (51.1%)	30 (73.2%)	27 (71.1%)	16 (72.7%)	<0.01
Arthritis	15 (11.3%)	17 (41.5%)	21 (55.3%)	11 (50.0%)	<0.01
Raynaud's phenomenon	46 (34.6%)	13 (31.7%)	12 (31.6%)	9 (40.9%)	0.85
Malignancy	16 (12.0%)	7 (17.1%)	5 (13.2%)	2 (9.1%)	0.93
**Disease activity**					0.16
Remission	34 (25.6%)	11 (26.8%)	6 (15.8%)	7 (31.8%)	
Low-to-moderate	85 (63.9%)	22 (53.7%)	21 (55.3%)	12 (54.5%)	
High	14 (10.5%)	8 (19.5%)	11 (28.9%)	3 (13.6%)	
**Number of CV risk factors**					<0.01
0	41 (30.8%)	1 (2.4%)	1 (2.6%)	1 (4.5%)	
1	44 (33.1%)	14 (34.1%)	6 (15.8%)	1 (4.5%)	
2	30 (22.6%)	14 (34.1%)	9 (23.7%)	5 (22.7%)	
≥3	18 (13.5%)	12 (29.3%)	22 (57.9%)	15 (68.2%)	
Arterial hypertension	44 (33.1%)	21 (51.2%)	23 (60.5%)	16 (72.7%)	<0.01
Hyperlipidaemia	59 (44.4%)	26 (63.4%)	26 (68.4%)	16 (72.7%)	<0.01
Diabetes mellitus	9 (6.8%)	4 (9.8%)	5 (13.2%)	5 (22.7%)	0.02
Established coronary artery disease	4 (3.0%)	12 (29.3%)	23 (60.5%)	18 (81.8%)	<0.01
History of myocardial infarction	5 (3.8%)	5 (12.2%)	10 (26.3%)	6 (27.3%)	<0.01
Family history of cardiovascular disease	45 (35.4%)	19 (47.5%)	18 (48.6%)	11 (50.0%)	0.07
**Autoantibodies**					
ANA	68 (51.1%)	21 (51.2%)	16 (42.1%)	8 (36.4%)	0.15
MSA	58 (43.6%)	16 (39.0%)	17 (44.7%)	11 (50.0%)	0.68
MAA	57 (42.9%)	20 (48.8%)	11 (28.9%)	10 (45.5%)	0.55
**Treatment**					
Glucocorticoid use	57 (42.9%)	12 (29.3%)	19 (50.0%)	7 (31.8%)	0.67

Variable	Absent CAC (n=133)	CAC mild (n=41)	CAC moderate (n=38)	CAC severe (n=22)	p value
Prednisolone-equivalent dose, mg/day	9.7 ± 12.1	10.2 ± 13.2	13.4 ± 16.9	7.7 ± 7.7	0.64
IVIg	22 (16.5%)	8 (19.5%)	8 (21.1%)	4 (18.2%)	0.60
**Number of DMARDs**					0.54
0	40 (30.1%)	17 (41.5%)	10 (26.3%)	5 (22.7%)	
1	71 (53.4%)	17 (41.5%)	26 (68.4%)	9 (40.9%)	
2	19 (14.3%)	5 (12.2%)	2 (5.3%)	8 (36.4%)	
≥3	3 (2.3%)	2 (4.8%)	0 (0.0%)	0 (0.0%)	

Supplementary Table S1. Clinical characteristics of patients with IIM stratified by CAC grade at the time of chest CT acquisition. Data are presented as n (%) or mean ± SD. *P* values refer to comparisons across CAC categories. Abbreviations: ANA, antinuclear antibodies; BMI, body mass index; CAC, coronary artery calcification; CV, cardiovascular; DMARD, disease-modifying antirheumatic drug; IIM, idiopathic inflammatory myopathy; IVIg, intravenous immunoglobulin; MAA, myositis-associated autoantibodies; MSA, myositis-specific autoantibodies; SD, standard deviation.

Model	HR for QRISK3 (per 1%-point increase) (95% CI)	HR for CAC (95% CI)	Harrell's C-index	AIC	LRT vs. QRISK3
**QRISK3 alone**	1.06 (1.03-1.10)	-	0.759	147.3	-
**CAC alone (any vs. none)**		10.15 (2.84-36.28)	0.784	140.6	-
**QRISK3 + at least mild CAC**	1.03 (0.99-1.07)	7.02 (1.76-27.99)	0.826	140.1	<0.01
**QRISK3 + at least moderate CAC**	1.01 (0.96-1.05)	17.98 (4.09-79.12)	0.870	130.5	<0.01
**QRISK3 + severe CAC**	1.04 (1.00-1.08)	11.94 (3.53-40.43)	0.847	134.7	<0.01

Supplementary Table S2. Additional prognostic value of visually assessed coronary artery calcification (CAC) beyond QRISK3 (1). Cox proportional hazards regression models were fitted for the composite endpoint of cardiovascular events (including non-fatal cardiac events, n=6) or cardiovascular death (n=11) occurring during follow-up after chest CT (mean 4.64 ± 4.60 years). Model discrimination was assessed using Harrell's C-index, model fit using the Akaike Information Criterion (AIC), and incremental prognostic value using likelihood-ratio tests (LRT) comparing each CAC model with the QRISK3-alone model.

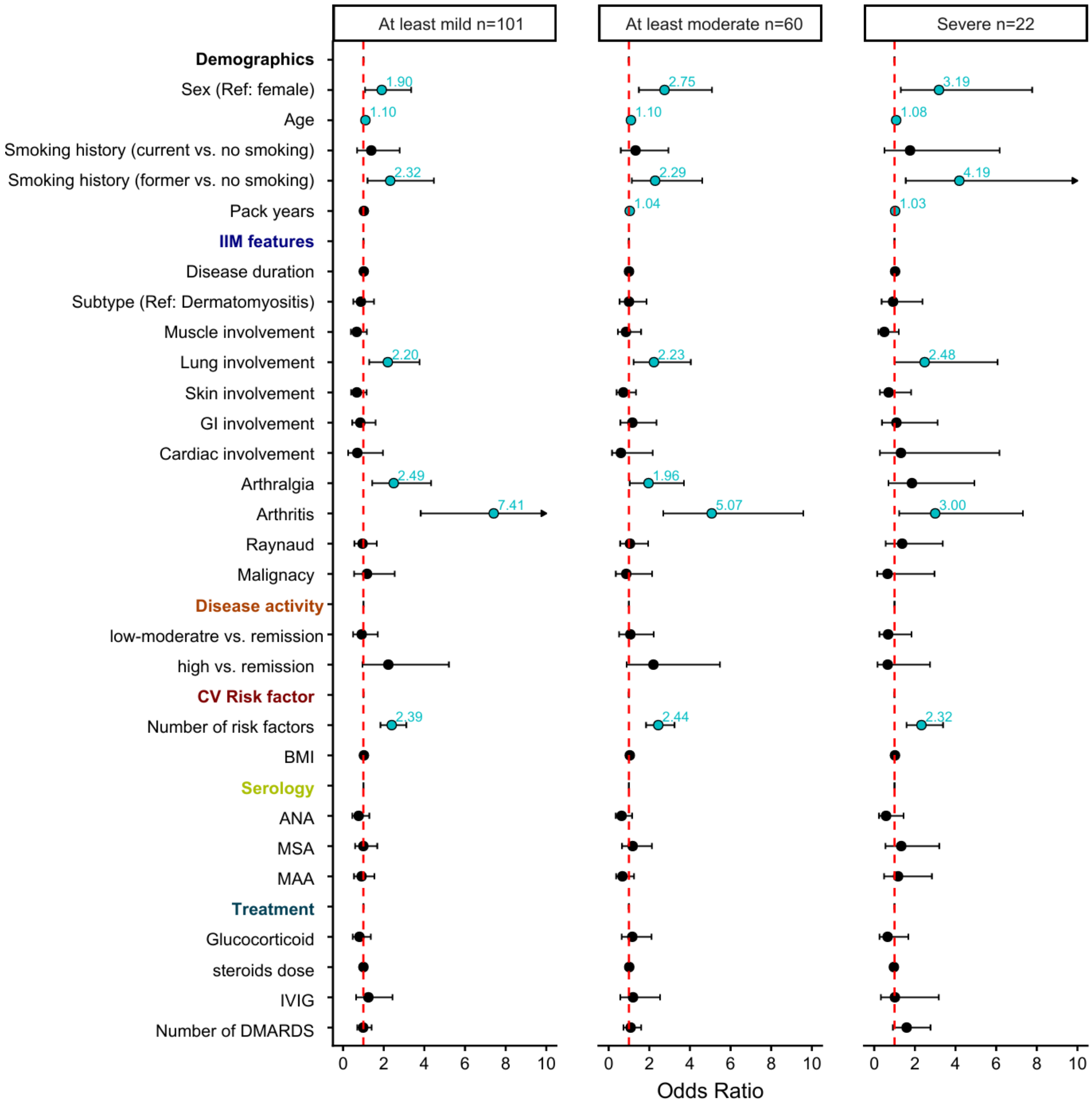

Supplementary Figure S2. Unadjusted odds ratios for clinical correlates of coronary artery calcification (CAC) severity in patients with IIM. Forest plots show odds ratios (ORs) with 95% confidence intervals (CIs) for clinical, serological, disease-related, and treatment-related variables associated with at least mild, at least moderate, and severe CAC on chest CT, each compared with no CAC as the reference category. Vertical dashed lines indicate OR = 1. Variables with statistically significant associations ($p < 0.05$) are highlighted in turquoise. Abbreviations: CAC, coronary artery calcification; CV, cardiovascular; CI, confidence interval; DMARDs, disease-modifying antirheumatic drugs; IIM, idiopathic inflammatory myopathies; MAA, myositis-associated antibodies; MSA, myositis-specific antibodies; IVIg, intravenous immunoglobulin, OR, odds ratio.

Variable	At least mild OR (95% CI)	At least moderate OR (95% CI)	Severe OR (95% CI)
Sex (Ref: female)	1.90 (1.08; 3.35)	2.75 (1.49; 5.08)	3.19 (1.31; 7.77)
Age	1.10 (1.07; 1.13)	1.10 (1.06; 1.13)	1.08 (1.04; 1.12)
Current smoking vs never	1.39 (0.69; 2.78)	1.32 (0.60; 2.94)	1.76 (0.50; 6.17)
Former smoking vs never	2.32 (1.20; 4.47)	2.29 (1.14; 4.60)	4.19 (1.55; 11.32)
Pack-years	1.02 (1.00; 1.05)	1.04 (1.01; 1.06)	1.03 (1.00; 1.06)
Disease duration	1.02 (0.98; 1.06)	1.00 (0.96; 1.05)	1.03 (0.97; 1.09)
IIM Subtype (Ref: Dermatomyositis)	0.88 (0.51; 1.52)	1.00 (0.54; 1.86)	0.93 (0.36; 2.38)
Muscle involvement	0.67 (0.38; 1.17)	0.85 (0.46; 1.60)	0.50 (0.20; 1.21)
Lung involvement	2.20 (1.29; 3.76)	2.23 (1.23; 4.04)	2.48 (1.01; 6.07)
Skin involvement	0.68 (0.40; 1.16)	0.73 (0.39; 1.34)	0.71 (0.28; 1.82)
GI involvement	0.85 (0.45; 1.60)	1.17 (0.58; 2.35)	1.09 (0.38; 3.12)
Cardiac involvement	0.70 (0.25; 1.96)	0.60 (0.17; 2.17)	1.31 (0.28; 6.16)
Arthralgia	2.49 (1.43; 4.33)	1.96 (1.04; 3.71)	1.86 (0.70; 4.93)
Arthritis	7.41 (3.82; 14.40)	5.07 (2.69; 9.58)	3.00 (1.23; 7.32)
Raynaud's syndrome	0.96 (0.56; 1.66)	1.05 (0.57; 1.94)	1.37 (0.56; 3.37)
Malignancy	1.18 (0.55; 2.54)	0.87 (0.35; 2.14)	0.66 (0.15; 2.97)
Disease activity: low-moderate vs remission	0.92 (0.49; 1.71)	1.07 (0.51; 2.22)	0.68 (0.25; 1.83)
Disease activity: high vs remission	2.23 (0.95; 5.21)	2.20 (0.89; 5.48)	0.66 (0.16; 2.74)
Number of CV risk factors	2.39 (1.84; 3.11)	2.44 (1.84; 3.24)	2.32 (1.59; 3.39)
Body mass index	1.03 (0.98; 1.08)	1.04 (0.99; 1.10)	1.02 (0.95; 1.10)
ANA positivity	0.77 (0.46; 1.29)	0.64 (0.35; 1.16)	0.58 (0.23; 1.45)
MSA positivity	1.00 (0.59; 1.68)	1.18 (0.66; 2.13)	1.33 (0.55; 3.20)
MAA positivity	0.91 (0.54; 1.54)	0.68 (0.37; 1.25)	1.17 (0.49; 2.84)
Current glucocorticoid use	0.80 (0.47; 1.37)	1.00 (1.00; 1.00)	0.66 (0.26; 1.68)
Prednisolone-equivalent dose	1.00 (0.98; 1.03)	1.01 (0.98; 1.04)	0.97 (0.90; 1.04)
IVIg	1.25 (0.64; 2.43)	1.20 (0.57; 2.53)	1.02 (0.33; 3.18)
Number of DMARDs	0.99 (0.70; 1.40)	1.08 (0.73; 1.60)	1.60 (0.92; 2.77)

Supplementary Table S3. Unadjusted odds ratios for clinical correlates of CAC severity in patients with IIM. Data are presented as odds ratio (95% confidence interval), with no CAC as the reference category.

Variable	At least mild OR (95% CI)	At least moderate OR (95% CI)	Severe OR (95% CI)
IIM Subtype (Ref: Dermatomyositis)	0.82 (0.42;1.60)	1.04 (0.50;2.18)	0.90 (0.32;2.54)
Muscle involvement	0.55 (0.28;1.11)	0.84 (0.40;1.79)	0.46 (0.17;1.25)
Lung involvement	1.84 (0.96;3.53)	1.71 (0.84;3.45)	1.83 (0.69;4.85)
Skin involvement	0.74 (0.38;1.42)	0.94 (0.45;1.95)	0.87 (0.31;2.46)
GI involvement	0.55 (0.26;1.19)	1.04 (0.46;2.36)	1.03 (0.33;3.25)
Cardiac involvement	0.66 (0.20;2.18)	0.60 (0.14;2.52)	1.62 (0.30;8.68)
Arthralgia	3.69 (1.81;7.53)	2.61 (1.20;5.67)	2.06 (0.70;6.02)
Arthritis	7.00 (3.21;15.27)	4.95 (2.31;10.59)	2.43 (0.92;6.45)
Raynaud's phenomenon	1.60 (0.80;3.21)	2.01 (0.91;4.43)	2.12 (0.75;5.99)
Malignancy	0.60 (0.24;1.51)	0.44 (0.15;1.25)	0.36 (0.07;1.80)
Disease activity: low-to-moderate vs remission	0.94 (0.42;2.08)	1.23 (0.50;3.05)	0.63 (0.21;1.92)
Disease activity: high vs remission	3.21 (1.12;9.23)	4.01 (1.29;12.50)	0.81 (0.17;3.77)
ANA positivity	1.09 (0.57;2.08)	0.92 (0.45;1.87)	0.84 (0.31;2.28)
MSA positivity	0.97 (0.51;1.85)	1.19 (0.59;2.39)	1.30 (0.50;3.39)
MAA positivity	1.26 (0.66;2.39)	0.84 (0.41;1.75)	1.72 (0.62;4.73)
Current glucocorticoid use	0.88 (0.46;1.67)	1.00 (1.00;1.00)	0.77 (0.28;2.16)
Prednisolone-equivalent dose	1.00 (0.97;1.04)	1.02 (0.99;1.05)	0.97 (0.90;1.05)
IVIg	1.18 (0.53;2.67)	1.20 (0.50;2.89)	1.12 (0.32;3.88)
Number of DMARDs	1.18 (0.74;1.87)	1.45 (0.87;2.41)	2.55 (1.27;5.11)

Supplementary Table S4. Adjusted odds ratios for clinical correlates of CAC severity in patients with IIM. Data are presented as odds ratio (95% confidence interval), with no CAC as the reference category. Models were adjusted for age, sex, disease duration and cardiovascular risk factor including smoking.

Variable	At least mild OR (95% CI)	At least moderate OR (95% CI)	Severe OR (95% CI)
IIM Subtype (Ref: Dermatomyositis)	0.83 (0.43;1.59)	1.05 (0.50;2.15)	0.93 (0.32;2.47)
Muscle involvement	0.56 (0.28;1.11)	0.84 (0.40;1.78)	0.48 (0.18;1.25)
Lung involvement	1.81 (0.95;3.46)	1.69 (0.84;3.38)	1.79 (0.70;4.69)
Skin involvement	0.74 (0.39;1.41)	0.94 (0.45;1.93)	0.89 (0.31;2.38)
GI involvement	0.56 (0.26;1.19)	1.05 (0.46;2.32)	1.08 (0.33;3.09)
Cardiac involvement	0.68 (0.20;2.13)	0.67 (0.15;2.39)	1.82 (0.31;7.59)
Arthralgia	**3.54 (1.79;7.30)**	**2.50 (1.19;5.51)**	1.93 (0.71;5.78)
Arthritis	**6.58 (3.14;14.56)**	**4.69 (2.26;10.08)**	2.33 (0.91;6.07)
Raynaud's phenomenon	1.57 (0.80;3.15)	1.95 (0.90;4.31)	2.06 (0.74;5.66)
Malignancy	0.61 (0.24;1.50)	0.46 (0.16;1.24)	0.43 (0.08;1.64)
Disease activity: low-to-moderate vs remission	0.93 (0.43;2.06)	1.20 (0.50;2.98)	0.62 (0.21;1.89)
Disease activity: high vs remission	**3.06 (1.11;8.85)**	**3.77 (1.27;11.83)**	0.86 (0.18;3.52)
ANA positivity	1.08 (0.57;2.06)	0.92 (0.46;1.85)	0.86 (0.32;2.22)
MSA positivity	0.98 (0.52;1.84)	1.18 (0.59;2.36)	1.28 (0.50;3.28)
MAA positivity	1.24 (0.66;2.36)	0.85 (0.41;1.73)	1.66 (0.62;4.51)
Current glucocorticoid use	0.88 (0.47;1.67)	1.63 (0.81;3.35)	0.79 (0.28;2.10)
Prednisolone-equivalent dose	1.00 (0.98;1.04)	1.02 (0.99;1.05)	0.99 (0.90;1.03)
IVIg	1.18 (0.53;2.63)	1.21 (0.50;2.83)	1.19 (0.33;3.65)
Number of DMARDs	1.18 (0.75;1.86)	1.43 (0.87;2.38)	**2.44 (1.26;4.89)**

Supplementary Table S5. Adjusted odds ratios from Firth penalized logistic regression models for clinical correlates of CAC severity in patients with IIM. Data are presented as odds ratio (95% confidence interval), with no CAC as the reference category. Results with $p<0.05$ are shown in bold.

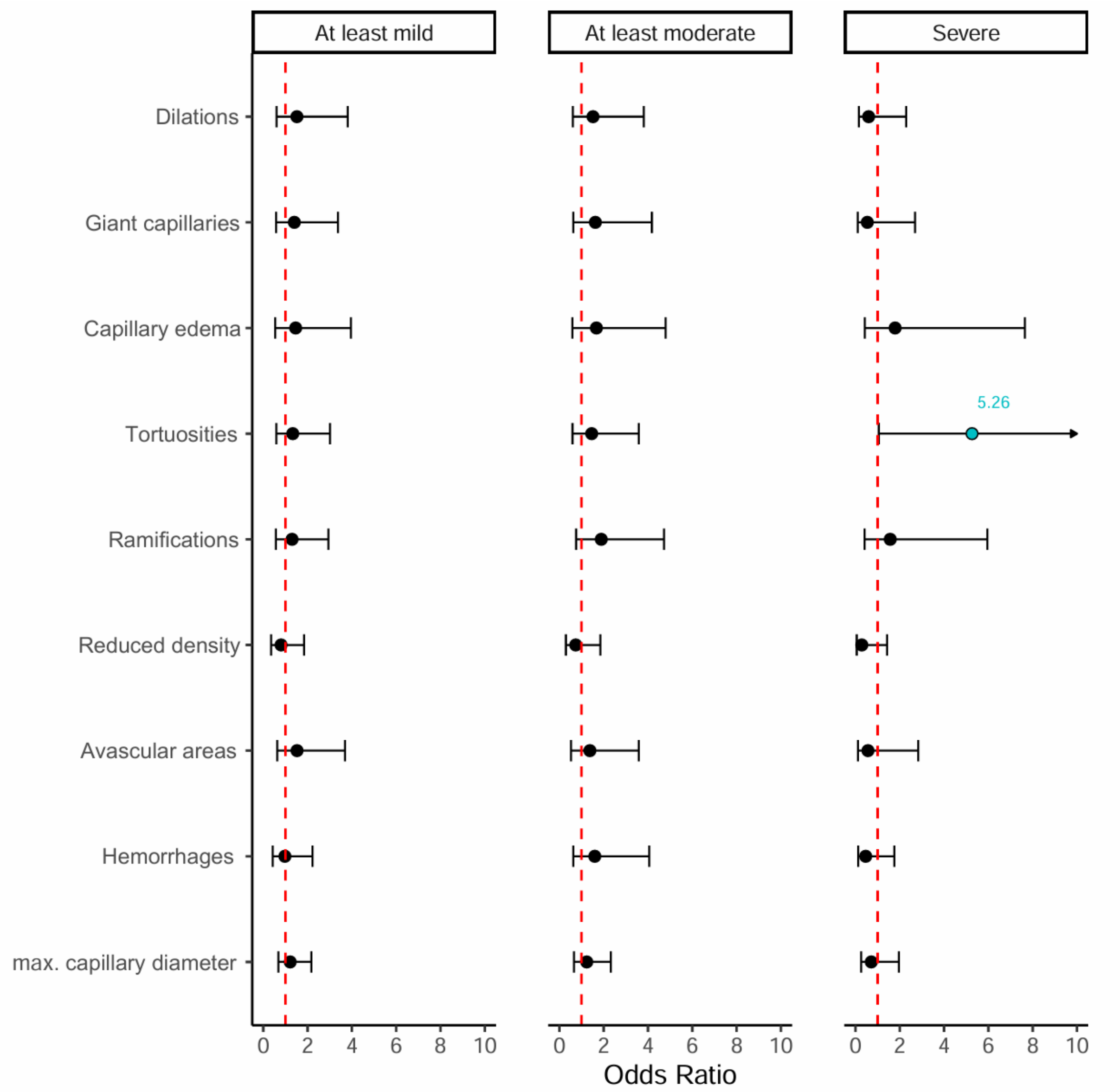

Supplementary Figure S3. Associations between nailfold capillaroscopic abnormalities and coronary artery calcification (CAC) severity in patients with IIM. Forest plots show odds ratios (ORs) with 95% confidence intervals (CIs) for microvascular features assessed within 6 months of the respective HRCT examination (available in n=103 patients)

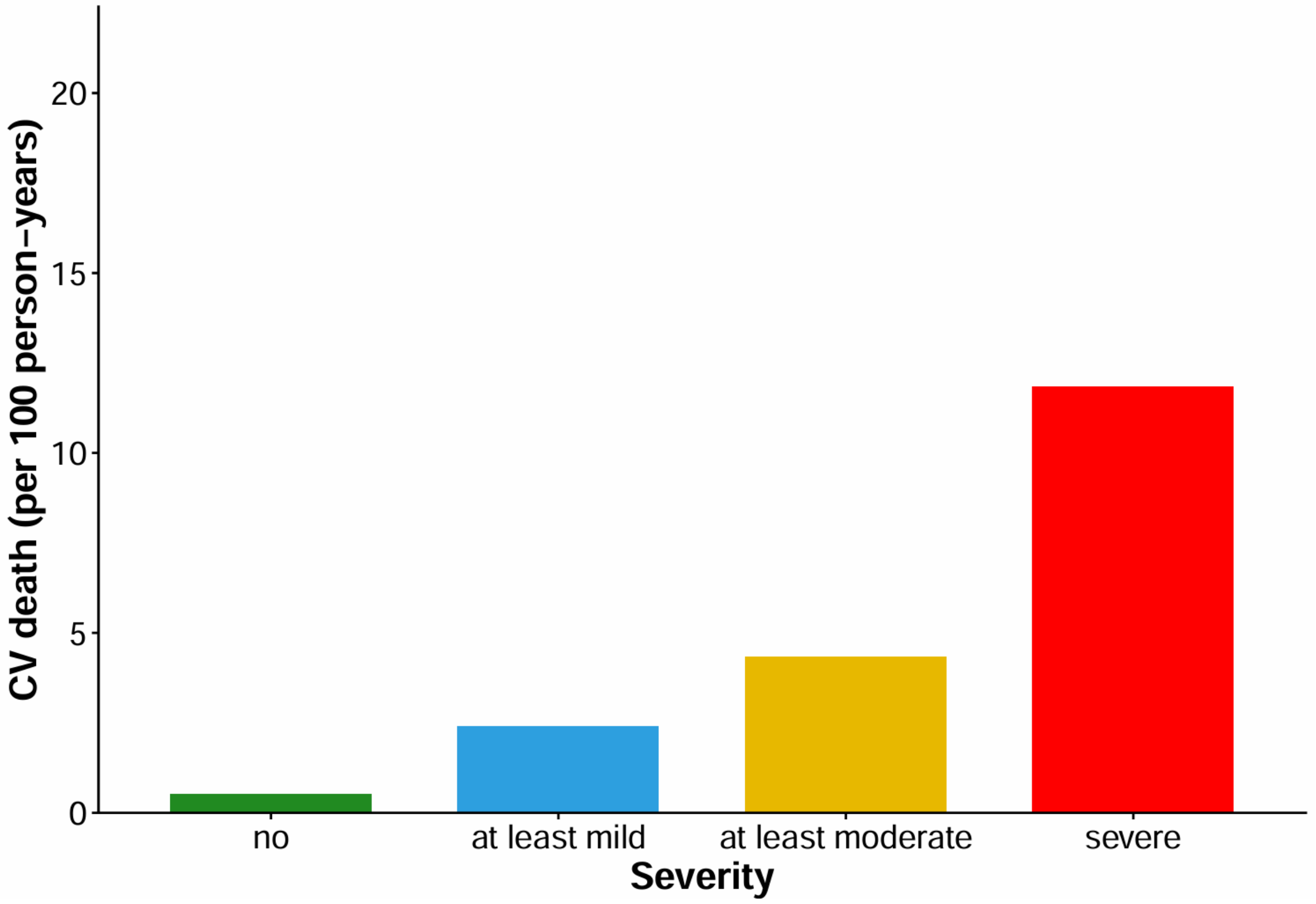

Supplementary Figure S4. CV-related death rates according to coronary artery calcification (CAC) category in patients with IIM. Bars indicate CV-related death rates per 100 person-years across CAC severity.

CAC category on chest CT	No. of events	Person-years	Rate per 100 person-years	95% CI
No/absent CAC	3	594.4	0.50	0.10;1.48
At least mild CAC	8	332.0	2.41	1.04;4.75
At least moderate CAC	8	184.4	4.34	1.87;8.55
Severe CAC	5	42.2	11.85	3.85;27.65

Supplementary Table S6. CV-related death according to coronary artery calcification (CAC) category, expressed as events per 100 person-years. Data are presented as number of events, person-years, and CV-related death rates per 100 person-years with 95% confidence intervals.

References:

1. Hippisley-Cox J, Coupland C, Brindle P. Development and validation of QRISK3 risk prediction algorithms to estimate future risk of cardiovascular disease: prospective cohort study. BMJ. 2017;357:j2099.
